# Identification of shared genetic susceptibility locus for coronary artery disease, type 2 diabetes and obesity: a meta-analysis of genome-wide studies

**DOI:** 10.1186/1475-2840-11-68

**Published:** 2012-06-14

**Authors:** Chaoneng Wu, Yunguo Gong, Jie Yuan, Hui Gong, Yunzeng Zou, Junbo Ge

**Affiliations:** 1 Shanghai Institute of Cardiovascular Diseases, Zhongshan Hospital, Fudan University, Shanghai 200032, China; 2 Institutes of Biomedical Sciences, Fudan University, 138 Yixueyuan Road, Shanghai 200032, China; 3 Shanghai Institute of Cardiovascular Diseases, Zhongshan Hospital, Fudan University, 180 Feng Lin Road, Shanghai 200032, China

**Keywords:** Meta-analysis, Type 2 diabetes, Obesity, Coronary artery disease, Genome-wide association study

## Abstract

Type 2 diabetes (2DM), obesity, and coronary artery disease (CAD) are frequently coexisted being as key components of metabolic syndrome. Whether there is shared genetic background underlying these diseases remained unclear. We performed a meta-analysis of 35 genome screens for 2DM, 36 for obesity or body mass index (BMI)-defined obesity, and 21 for CAD using genome search meta-analysis (GSMA), which combines linkage results to identify regions with only weak evidence and provide genetic interactions among different diseases. For each study, 120 genomic bins of approximately 30 cM were defined and ranked according to the best linkage evidence within each bin. For each disease, bin 6.2 achieved genomic significanct evidence, and bin 9.3, 10.5, 16.3 reached suggestive level for 2DM. Bin 11.2 and 16.3, and bin 10.5 and 9.3, reached suggestive evidence for obesity and CAD respectively. In pooled all three diseases, bin 9.3 and 6.5 reached genomic significant and suggestive evidence respectively, being relatively much weaker for 2DM/CAD or 2DM/obesity or CAD/obesity. Further, genomewide significant evidence was observed of bin 16.3 and 4.5 for 2DM/obesity, which is decreased when CAD was added. These findings indicated that bin 9.3 and 6.5 are most likely to be shared by 2DM, obesity and CAD. And bin 16.3 and 4.5 are potentially common regions to 2DM and obesity only. The observed shared susceptibility regions imply a partly overlapping genetic aspects of disease development. Fine scanning of these regions will definitely identify more susceptibility genes and causal variants.

## Introduction

Type 2 diabetes (2DM), obesity, and coronary artery disease (CAD) are frequently coexisting disorders and major components of metabolic syndrome that cause a substantial public health and economic burden worldwide. Susceptibility to such complex diseases is strongly influenced by multiple genetic factors combined with environmental factors, and all these diseases are further characterized by a chronic inflammatory process. The strong genetic basis has been successfully revealed for each of these diseases. However, whether there are shared or interactive genetic background underlying all three diseases has remained largely unknown.

 Actually, evidence has showed that some loci confer risk for more than one of the studied diseases, and most common diseases arise from interaction between multiple genetic variations. This point suggested the concept of common genetic underpinnings for common diseases [1]. For example, a Wellcome Trust Case Control Consortium study evaluated 3,000 shared controls and 2,000 cases for each of seven complex human diseases—bipolar disorder, CAD, Crohn’s disease, hypertension, rheumatoid arthritis, type 1 diabetes, and 2DM—and demonstrated common susceptibility regions for rheumatoid arthritis and type 1 diabetes [2]. The common genetic basis for 2DM and obesity has also been indicated, with the common obesity genes being found through 2DM studies [3]. Further, CAD and 2DM have also been suggested to spring from shared genetic effects, rather than CAD being a complication of diabetes. These data indicates the potentially strong common genetic aspects among 2DM, obesity and CAD.

Genome-wide association studies (GWAS) is one recent revolution, in which hundreds and thousands of single nucleotide polymorphisms (SNPs) are genotyped to capture indirectly most of the genome’s common variation [4]. Compared to the hypothesis-driven linkage and candidate-gene studies, GWAS is unbiased with regard to presumed functions or locations of causal variants. A series of GWA investigations have been reported in 2DM, obesity and CAD recently, with some of them being robustly replicated in large sample sizes. These ever fast-growing numbers of the GWA studies offer the opportunity to combine the recent findings across different investigations, so as to increase the statistical power and to identify the regions with smaller effects. Furthermore, the combination of data across differently interactive diseases might reveal the potentially underlying shared gentic mechanisms.

For this aim, we performed a meta-analysis of autosomal genome GWA scans of 35 2DM studies, 36 studies of obesity or BMI-defined obesity, and 22 CAD studies by using genome search meta-analysis (GSMA) method, an effective and convient nonparametric method to combine results across the genome [5].

## Materials and methods

### Eligible genome scans

The studies were identified by an extended search of the the database of GWS ( http://www.genome.gov/gwastudies) and PubMed database. In PubMed, we used the following terms: (linkage AND genome-scan OR genome OR genomewide OR genome-wide OR LOD ((logarithm of odds) OR microsatellite) combined with (coronary artery disease OR coronary heart disease), or with (obesity OR body mass index OR body mass index OR BMI), or with (2DM OR type 2 diabetes OR type 2 diabetic mellitus). The limits we set included publication in English, human studies, 1998–2012, and the exclusion of reviews. Only primary GWAS were included. Excluded were “fine mapping” with additional markers or pedigrees in selected regions, as well as scans restricted to specific individual chromosome or chromosomal regions. Some studies with genetic mapping figures not available or studies whose markers were difficult to place on the current map were discarded. For a relatively homogenous phenotype, studies of coronary artery calcification or studies about obesity traits (such as leptin or other anthropometric measures) were discarded either.

### Data extraction

For each eligible study, the following information was extracted: first author with year of publication, racial descent of study population, number of families and affected individuals, numbers of case–control subjects, number of markers, and types of statistical analysis. Genome scan data across each chromosome were derived from the figures provided in the published papers after digitization and from the results presented in each study.

### GSMA method

GSMA is a nonparametric method for combining results from across the genome that were generated with different maps and statistical tests. It does not require individual-level genotype data. As a rank-based meta-analysis method, GSMA assesses the strongest evidence for linkage within prespecified genomic regions, termed “bins”. Briefly, the genome was divided into 120 bins of approximately 30 cM. Bins are referred to by chromosome, so “bin 1.4” indicates the fourth bin on chromosome 1. Each graph from the published GWA studies was imported into CorelDraw and overlaid with a grid dividing each chromosome equally into the required number of bins. Then the peak height within each bin was measured using a graphical ruler within the drawing program, and the maximum linkage statistic within a bin was identified (e.g., maximum LOD or NPL score (Nonparametric multipoint linkage), or minimum *P*-value). Bins were then ranked, and for each bin, ranks (or weighted ranks) were summed across studies, with the summed rank (SR) forming a test statistic. The significance of the SR in each bin was assessed using Monte Carlo simulations, permuting the bin location of ranks within each study to obtain an empirical *P*-value.

Analysis was performed using the GSMA software ( http://www.kcl.ac.uk/ mmg/ research/gsma/), with 10,000 simulations and nominal significance. The traditional 30-cM bin definition gives a total of 120 bins on the basis of the Marshfield genetic map ( http://www.bli.uzh.ch/BLI/Projects/genetics/maps/marsh.html). To control for multiple testing, we used a Bonferroni correction for the number of bins across the genome: for 30-cM bins, a *P*-value of 0.00042 (0.05/120 = 0.00042) was necessary for genome-wide significant evidence of linkage, and a *P*-value of 1/120 = 0.0083 for suggestive evidence of linkage. Corresponding *P*-values were calculated for other bin sizes. Considering the different number of cases for the three diseases, the number of cases of each disease was weighted, with each *R* study value being multiplied by its study’s weight $\left(\sqrt{N\left[\text{affected}\;\text{case}\right]}\right)$, divided by the mean of this value over all studies, as discussed firstly by Levinson et al [6].

## Results

### Data included

The selection criteria and data requests provided 35 studies for 2DM comprising 378,132 samples (4,532 pedigree, 42,200 cases, 86,253 controls) (Additional file 1: Table S1); 36 studies for obesity or BMI-defined obesity comprising 358,860 samples (9,973 pedigree, 348,887 subjects) (Additional file 1: Table S2); and 21 CAD studies comprising 191,126 samples (2,036 pedigree, 69,828 case, 119,262 control) (Additional file 1: Table S3).

### Linkage evidence for each disease

**Figure 1:**
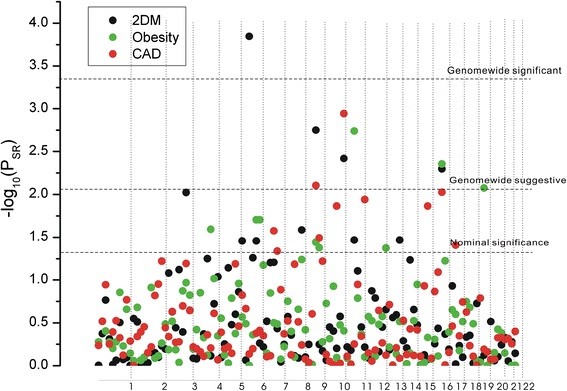
**Linkage evidence for each disease.** The *-log*_*(10) *_*P* value of the summed rank (*P*_*SR*_) for each bin was shown. The dashed lines represent three different thresholds level: genome-wide significance (using a Bonferroni correction for testing 120 bins, *P*_*SR*_ = 0.05/120 = 0.00042), suggestive significance level (*P*_*SR*_ = 1/120 = 0.0083), and nominal significance (*P*_*SR*_ = 0.05).

For linkage evidence of 2DM, bin 6.2 reached genome-wide significance (*P*_*SR*_ = 0.000143), with two adjacent bins (6.4 and 6.5) showing nominal significance (Figure 1, Table 1). Three other bins, 9.3, 10.5, 16.3, also reached suggestive level (*P*_*SR*_ = 0.001767, *P*_*SR*_ = 0.003829 and *P*_*SR*_ = 0.005043, respectively) (Figure 1, Table 1). Bin 8.5 and 11.2 showed nominal significance (Figure 1, Table 1).

**Table 1:** Linkage evidence for each disease of 2DM, obesity and CAD

	**2DM**			**Obesity**			**CAD**		
**Pos**	**Bin**	**Cytogenetic band**	***P***_**SR**_	**Bin**	**Cytogenetic band**	***P***_**SR**_	**Bin**	**Cytogenetic band**	***P***_**SR**_
1	6.2	6p22.3-p21.1	0.000143	11.2	11p15.1-p12	0.001822	10.5	10q23.33-q26.13	0.001135
2	9.3	9p21.1-q21.32	0.001767	16.3	16q12.2-q23.1	0.004428	9.3	9p21.1-q21.32	0.007895
3	10.5	10q23.33-q26.13	0.003829	19.3	19q12-q13.33	0.008426	16.3	16q12.2-q23.1	0.009467
4	16.3	16q12.2-q23.1	0.005043	6.5	6q23.2-q25.3	0.018402	11.5	11q22.3-q24.1	0.011467
5	3.6	3q22.1-q25.31	0.009509	6.4	6q15-q23.2	0.019753	15.3	15q21.3-q26.1	0.013732
6	8.5	8q22-q24.21	0.026031	4.5	4q24-q28.3	0.025605	7.3	7p14.1-q21.11	0.026741
7	11.2	11p15.1-p12	0.034162	9.3	9p21.1-q21.32	0.036314	9.4	9q21.32-q31.1	0.032157
8	6.4	6q15-q23.2	0.035033	9.4	9q21.32-q31.1	0.042022	7.4	7q12.11-q31.1	0.046022

2DM, type 2 diabetes; CAD, coronary artery disease.

For obesity, bin 11.2 and 16.3 reached suggestive level (*P*_*SR*_ = 0.001822 and *P*_*SR*_ = 0.004428, respectively, Figure 1, Table 1). And six other bins (19.3, 6.3, 6.4, 4.5, 9.3 and 9.4) showed nominal significance (Figure 1, Table 1). For CAD, two bins, 10.5 and 9.3, reached the suggestive level (*P*_*SR*_ = 0.001135 and *P*_*SR*_ = 0.007895, respectively). And six other bins,16.3, 11.5, 15.3, 7.3, 9.4 and 7.4, showed nominal significance (Figure 1, Table 1).

### Linkage evidence for combined each two or all three diseases

**Figure 2:**
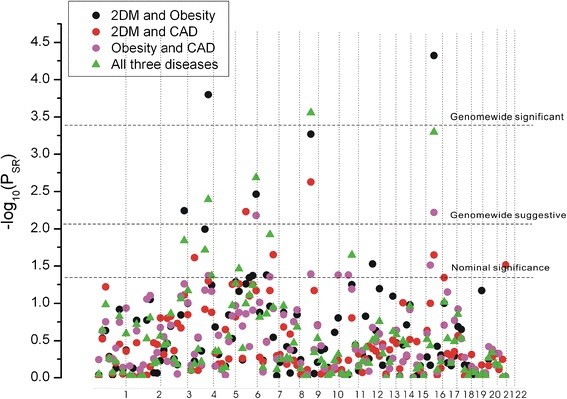
**Linkage evidence for combined each two or all three diseases.** The *-log*_*(10) *_*P* value of the summed rank (*P*_*SR*_) for each two and all three diseases was shown. The dashed lines represent three different thresholds level: genome-wide significance (using a Bonferroni correction for testing 120 bins, *P*_*SR*_ = 0.05/120 = 0.00042), suggestive significance level (*P*_*SR*_ = 1/120 = 0.0083), and nominal significance (*P*_*SR*_ = 0.05).

In pooled analysis, bin 9.3 and bin 6.5 were most likely to be shared by all the three diseases. Bin 9.3 reached suggestive evidence for 2DM/CAD (*P*_*SR*_ = 0.002362) and 2DM/obesity (*P*_*SR*_ = 0.000543), and normial evidence for CAD/obesity (*P*_*SR*_ = 0.0405) (Figure 2, Table 2). However, this region achieved genomewide significant evidence in pooled all the three diseases (*P*_*SR*_ = 0.000276). Bin 6.5 reached suggestive evidence for 2DM/obesity (*P*_*SR*_ = 0.003457) and CAD/obesity (*P*_*SR*_ = 0.006592), yet no significant evidence for 2DM/CAD (*P*_*SR*_ = NS) (Figure 2, Table 2). Still, in pooled all the three disease, this region showed suggestive evidence with a much smaller value of *P*_*SR*_ (*P*_*SR*_ =0.00205) (Figure 2, Table 2).

**Table 2:** Linkage evidence for combined each two or all three diseases of 2DM, obesity and CAD

	**2DM/CAD**			**2DM/Obesity**		
**Pos**	**Bin**	**Cytogenetic band**	***P***_**SR**_	**Bin**	**Cytogenetic band**	***P***_**SR**_
1	9.3	9p21.1-q21.32	0.002362	16.3	16q12.2-q23.1	4.75E-05
2	6.2	6p22.3-p21.1	0.005861	4.5	4q24-q28.3	0.000159
3	7.4	7q12.11-q31.1	0.022381	9.3	9p21.1-q21.32	0.000543
4	16.3	16q12.2-q23.1	0.022471	6.5	6q23.2-q25.3	0.003457
5	4.1	4pter-p15.33	0.024538	2.3	2p23.2-p16.2	0.00572
6	22.2	22q12.3-pter	0.030478	4.4	4q13.3-q24	0.010083
7	17.2	17p12-q21.33	0.045176	12.3	12p11.21-q15	0.029702
	**CAD/Obesity**			**All three diseases**		
**Pos**	**Bin**	**Cytogenetic band**	***P***_**SR**_	**Bin**	**Cytogenetic band**	***P***_**SR**_
1	16.3	16q12.2-q23.1	0.006016	9.3	9p21.1-q21.32	0.000276
2	6.5	6q23.2-q25.3	0.006592	16.3	16q12.2-q23.1	0.000505
3	16.2	16p13-q12.2	0.030775	6.5	6q23.2-q25.3	0.002055
4	9.3	9p21.1-q21.32	0.040512	4.5	4q24-q28.3	0.004021
5	10.5	10q23.33-q26.13	0.041678	7.3	7p14.1-q21.11	0.011893
6	11.2	11p15.1-p12	0.041838	3.6	3q22.1-q25.31	0.014341
7	4.5	4q24-q28.3	0.042347	4.4	4q13.3-q24	0.019338
8	7.3	7p14.1-q21.11	0.043952	11.3	11p12-q13.3	0.022461

2DM, type 2 diabetes; CAD, coronary artery disease.

Bin 16.3 and bin 4.5 were most likely to be shared by 2DM and obesity only. In 2DM/obesity, these two bins achieved genomewide significant evidence (*P*_*SR*_ = 4.75E-05 and 0.000159, respectively) (Figure 2, Table 2). For bin 16.3, it showed norminal evidenc for 2DM/CAD and suggestive evidence for CAD/Obesity (*P*_*SR*_ = 0.022471 and 0.006016, respectively) (Figure 2, Table 2). For bin 4.5, it showed norminal evidence for CAD/obesity (*P*_*SR*_ = 0.042347) and no significant evidence for 2DM/CAD (*P*_*SR*_ = NS) (Figure 2, Table 2). In pooled all three diseases, the evidence decreased to be suggestive level for either bin 16.3 or bin 4.5 (*P*_*SR*_ =0.000505 and 0.004021, respectively) (Figure 2, Table 2).

## Discussion

2DM and obesity have long been recognized as major risk factors for CAD. In addition, the development of CAD can also follow or precede 2DM or obesity. Strong genetic factors for each disease have been recognized in previous studies. However, the common genetic aspects underlying all these three diseases remain undetermined. Here, we provided evidence that bin 9.3 and 6.5 were potentially common genetic regions to all the three diseases, and bin 16.3 and 4.5 were most likely to be shared by only 2DM and obesity.

### Bin 9.3 (9p21.1-q21.32)

Bin 9.3 achieved much stronger evidence for pooled analysis of all the three diseases compared to each two diseases combination. The increased stastical power for this region suggested a mutually reinforcing interaction among the 2DM, obesity and CAD. Within this region, 9p21.3 is a loci approximately 22 million base pairs from the 9p telomere. This region was firstly identified to be associated with CAD across different ethinics. The genetic risk variant from this region is extremely common, with 75% of the Caucasian population having one or more risk alleles. In CAD, the locus is heterozygous in 50% of Caucasians and homozygous in 25% with increased risk of 15–20% and 30–40% respectively. A series of studies have reported that this region is associated with the risk for 2DM [7-11]. Evidence can also be found that the variants of 9p21.3 are implicated in obesity [12].

The region of 9p21 maps two well-characterized tumor suppressor genes, *CDKN2A*/ *CDKN2B*, encoding respectively proteins p16 ^INK4a^ and p15 ^INK4b^, both of them are involved in the regulation of cell proliferation, cell aging and apoptosis. Protein p16^INK4a^ inhibits cyclin-dependent kinase 4(CDK4) and is a strong regulator of pancreatic beta cell replication [13,14]. In addition, p16^INK4a^ can be an important regulator for chronic inflammation, and deficiency of p16^INK4a^ results in decreased inflammatory signaling in murine macrophages and influences the phenotype of human adipose tissue macrophages [15,16]. It has been suggested that chronic inflammatory process is implied in CAD, 2DM and obesity. It is therefore these data supports that defects in 9p21.3 might be the common genetic factors, indicating a chronic inflammation process that predispose to the sequelae of 2DM, obesity and CAD.

### Bin 6.5 (6q23.2-q25.3)

Bin 6.5 reached much stronger evidence for the linkage of all the three diseases compared to the combination to each two diseases, implying the potentially common gentic aspects for all the three diseases of bin 6.5.

The gene *ENPP1* (ectonucleotide pyrophosphate phosphodiesterase), which encodes a membrane-bound glycoprotein inhibiting the insulin-receptor tyrosine kinas activity and reducing insulin sensitivity, appears to be one of the candidate genes in the region. Evidence can be found that the variants of *ENPP1* were associated with insulin resistance (IR)/atherogenic phenotypes, including earlier onset of 2DM and myocardial infarction. And it is also associated with the genetic susceptibility for metabolic syndrome in CAD patients [17,18]. The report by Bacci S, et al., showed the variant of *ENPP1* is an independent predictor of major cardiovascular events, and this effect is exacerbated by obesity in 2DM patients [19]. A set of studies have also suggested that the variant of *ENPP1* were associated with only obesity-type 2DM, which indicating the substantial etiological heterogeneity of 2DM mediated by the obesity status based on the shared genetic loci [20-22]. Elucidation of the interplay of *ENPP1* in increased susceptibility to 2DM, obesity and CAD will provide recommendations for the underlying shared mechanisms of these complex common diseases.

### Bin 16.3 (16q12.2-q23.1)

Bin 16.3 was the most significant region for linkage to 2DM/obesity, with much decreased evidence for pooled analysis of all the three diseases, indicating the common genetic aetiology for 2DM/obesity of bin 16.3. One most interesting gene from this region is *FTO* (fat mass and obesity associated), encoding 2-oxoglutarate-dependent nucleic acid demethylase. *FTO* harbours the strongest known obesity-susceptibility locus. In animal models, knock-out of *FTO* resulted in growth retardation, loss of white adipose tissue, and increase energy metabolism and systemic sympathetic activation. In contrast, *FTO* overexpression results in a dose-dependent increase in BMI and develop glucose intolerance on high-fat diet [23]. Amounting evidence has also suggested that *FTO* is associated with metabolic profiles including dyslipidemia and insulin resistance, and increased the risk for 2DM [24-28]. Although multiple association studies as well as functional experiments have revealed that *FTO* is critical for obesity and 2DM, only one study with really small sample size has reported that *FTO* is assocaited with increased rifk for acute coronary syndrome, a severe form of CAD [29]. Our results did not support that this region was shared by 2DM/obesity and CAD. Even though, our findings suggested the underlying shared genetic region of bin 16.3, which is worthy of further investigation.

### Bin 4.5 (4q24-q28.3)

Bin 4.5 was also worthy of note when deciphering the common susceptibility loci. Bin 4.5 achieved genomewide significance evidence for 2DM/obesity, which become much weaker in pooled analysis of all three diseases, indicating this loci being shared by 2DM/obesity but divergent from CAD.

*FABP2* might be a potential common candidate gene from this region. *FABP2* (fatty acid binding protein 2) is an intracellular proteins expressed only in the intestine and involved in the absorption and intracellular transport of dietary long-chain fatty acids. The association of *FABP2* with both 2DM and obesity has been reported by several studies [30-33]. Previous reports showed that the variants of *FABP2* increased flux of dietary fatty acids into the circulation, and was also associated with increased fasting inculin concentration, fasting fatty acid oxidation and reduced glucose uptake, all are etiology of metabolic disorders [34]. In our study, this region was shared by 2DM/obesity but not CAD. The report by Georgopoulos A et al., showed that the variant of *FABP2* increase the cardiovascular risk in dyslipidemic men with diabetes compared to their non-diabetic counterparts with 2 ~ 3.5-fold, which indicates the influence of the variant of *FABP2* to lipid and glucose metabolic disorders and then to affect the risk to CAD [35]. Larger studies focusing on analysis of *FABP2* in patients with 2DM and obesity will augment our preliminary results.

### Other regions

Here, bin 19.3 reached suggestive evidence for independent obesity analysis. One most reported gene from this region is Apolipoprotein E (*APOE*), which influences on lipid profiles and is associated with the development of both 2DM with and without CAD, and furthermore, it increased the risk among the subjects with obesity and/or smoking, conditions that are associated with high oxidative stress [36]. The expression of *APOE* can be regulated by peroxisome proliferator-activated receptor gamma (*PPARG*, bin 3.1), a potential common gene for glucose and lipid metabolism as well as CAD development [37,38]. In addition, *PPARG* interactes with another transfaction factor of forkhead box protein O1 (*FOXO1*, bin 13.4) to regulate the expression of mitochondrial uncoupling protein 2 (*UCP2*, bin11.4) and beta-3 adrenergic receptor (*ADRB3*, bin 8.3), all these genes being associated with metabolic disturbances such as obesity and 2DM [39-41]. These data collectively indicated the potential complex gene interactions for these closely related disorders.

## Conclusions

In summary, our results provide evidence that bin 9.3 and 6.5 are most likely to be shared by 2DM, obesity and CAD. Bin 16.3 and 4.5 were potentially common regions to 2DM and obesity only. The observed shared susceptibility regions for each two or all the three diseases suggest a common genetic cause for these diseases which implies a partly overlapping genetic aspects of disease development. Eventhough it remained unclear that one disease may have a direct causal influence on the susceptibility of the other, the potential interactive regions we identified here await for further elucidation. Fine scans of these regions will definitely identify more susceptibility genes and causal variants for these common diseases.

### Electronic-database information

Accession Numbers and URLs for data in this article are as follows:

Online Mendelian Inheritance in Man (OMIM), http://www.ncbi.nlm.nih.gov/Omim

Marshfield genetic map, http://research.marshfieldclinic.org/genetics/home/index.asp

GSMA software, http://www. kcl.ac.uk/mmg/research/gsma/ [42–133].

## Abbreviations

2DM: Type 2 diabetes; CAD: Coronary artery disease; GWAS: Genome-wide association studies; SNPs: Single nucleotide polymorphisms; GSMA: Genome search meta-analysis; BMI: Body mass index; NPL: Nonparametric multipoint linkage; MLS: Maximum LOD score; LOD: Logarithm of odds; ZLR: Ikelihood ratio z-score; SR: Summed rank; CDK4: Cyclin-dependent kinase 4; ENPP1: Ectonucleotide pyrophosphate phosphodiesterase; IR: Insulin resistance; FTO: Fat mass and obesity associated; FABP2: Fatty acid binding protein 2; APOE: Apolipoprotein E; PPARG: Peroxisome proliferator-activated receptor gamma; FOXO1: Forkhead box protein O1; UCP2: Mitochondrial uncoupling protein 2; ADRB3: Beta-3 adrenergic receptor.

## Competing interests

The authors declare that they have no conflicts of interest.

## Authors’ contributions

Chaoneng Wu and Yunguo Gong participated in designing, carried out the literature reviewing and data stastical analysis, and drafting the manuscript. Jie Yuan and Hui Gong participated in literature reviewing and data extraction. Yunzeng Zou and Junbo Ge conceived of the study, and participated in its design and coordination and helped to draft the manuscript. All authors read and approved the final manuscript.

## Supplementary Material

Additional file 1 **Table S1.** Characteristics of whole genome studies of T2D [42–75,2]. **Table S2.** Characteristics of whole genome studies of obesity or BMI-defined obesity [76–111]. **Table S3.** Characteristics of whole genome studies of CAD [112–132]. Click here for file
