# Supplementary material for: Identification of shared genetic susceptibility locus for coronary artery disease, type 2 diabetes and obesity: a meta-analysis of genome-wide studies
Source: Cardiovasc Diabetol. 2012 Jun 14;11:68. doi: 10.1186/1475-2840-11-68 (PMC3481354; doi:10.1186/1475-2840-11-68)
Supplement: Additional file 1 — Table S1. Characteristics of whole genome studies of T2D [42–75,2]. Table S2. Characteristics of whole genome studies of obesity or BMI-defined obesity [76–111]. Table S3. Characteristics of whole genome studies of CAD [112–132]. [file 1475-2840-11-68-S1.doc]

Table S1. Characteristics of whole genome studies of T2D

| First author (year) | Population | Phenotype | Ped^a^ | Aff^b^ | Case | Control | Markers | Statistics |
| --- | --- | --- | --- | --- | --- | --- | --- | --- |
| [Palmer (2012)[42]](../../../../C:%5CDocuments%20and%20Settings%5CAdministrator%5C%E6%A1%8C%E9%9D%A2%5CBook1.xls#RANGE!_ENREF_1%23RANGE!_ENREF_1) | African American | 2DM |  |  | 965 | 1029 | 832,357 | *P*-value |
| [Cui (2011)[43]](../../../../C:%5CDocuments%20and%20Settings%5CAdministrator%5C%E6%A1%8C%E9%9D%A2%5CBook1.xls#RANGE!_ENREF_2%23RANGE!_ENREF_2) | Chinese (Han) | 2DM |  |  | 793 | 806 | 474,515 | P-value |
| [Cho (2011)[44]](../../../../C:%5CDocuments%20and%20Settings%5CAdministrator%5C%E6%A1%8C%E9%9D%A2%5CBook1.xls#RANGE!_ENREF_3%23RANGE!_ENREF_3) | Asian | 2DM |  |  | 5843 | 4574 | 2,626,356 | *P*-value |
| [Kooner (2011)[45]](../../../../C:%5CDocuments%20and%20Settings%5CAdministrator%5C%E6%A1%8C%E9%9D%A2%5CBook1.xls#RANGE!_ENREF_4%23RANGE!_ENREF_4) | South Asian | 2DM |  |  | 5561 | 14458 | 568,976 | *P*-value |
| [Below (2011)[46]](../../../../C:%5CDocuments%20and%20Settings%5CAdministrator%5C%E6%A1%8C%E9%9D%A2%5CBook1.xls#RANGE!_ENREF_5%23RANGE!_ENREF_5) | Mexican-American | 2DM |  |  | 837 | 436 | 1,829,586 | *P*-value |
| [Tsai (2010)[47]](../../../../C:%5CDocuments%20and%20Settings%5CAdministrator%5C%E6%A1%8C%E9%9D%A2%5CBook1.xls#RANGE!_ENREF_6%23RANGE!_ENREF_6) | Chinese (Han) | 2DM |  |  | 995 | 894 | 516,737 | *P*-value |
| [Shu (2010)[48]](../../../../C:%5CDocuments%20and%20Settings%5CAdministrator%5C%E6%A1%8C%E9%9D%A2%5CBook1.xls#RANGE!_ENREF_7%23RANGE!_ENREF_7) | Chinese (Han) | 2DM |  |  | 1019 | 1710 | 590,887 | *P*-value |
| [Yamauchi (2010)[49]](../../../../C:%5CDocuments%20and%20Settings%5CAdministrator%5C%E6%A1%8C%E9%9D%A2%5CBook1.xls#RANGE!_ENREF_8%23RANGE!_ENREF_8) | Japanese | 2DM |  |  | 4470 | 3071 | 459,359 | *P*-value |
| [Voight (2010)[50]](../../../../C:%5CDocuments%20and%20Settings%5CAdministrator%5C%E6%A1%8C%E9%9D%A2%5CBook1.xls#RANGE!_ENREF_9%23RANGE!_ENREF_9) | European | 2DM |  |  | 8130 | 38987 | 2,426,886 | *P*-value |
| [Qi (2010)[51]](../../../../C:%5CDocuments%20and%20Settings%5CAdministrator%5C%E6%A1%8C%E9%9D%A2%5CBook1.xls#RANGE!_ENREF_10%23RANGE!_ENREF_10) | European | 2DM |  |  | 2591 | 3052 | European | *P*-value |
| [Rung (2009)[52]](../../../../C:%5CDocuments%20and%20Settings%5CAdministrator%5C%E6%A1%8C%E9%9D%A2%5CBook1.xls#RANGE!_ENREF_11%23RANGE!_ENREF_11) | French | 2DM |  |  | 679 | 697 | 392,365 | *P*-value |
| [Takeuchi (2009)[53]](../../../../C:%5CDocuments%20and%20Settings%5CAdministrator%5C%E6%A1%8C%E9%9D%A2%5CBook1.xls#RANGE!_ENREF_12%23RANGE!_ENREF_12) | Japanese | 2DM |  |  | 519 | 530 | 482,625 | *P*-value |
| [Sale (2009)[54]](../../../../C:%5CDocuments%20and%20Settings%5CAdministrator%5C%E6%A1%8C%E9%9D%A2%5CBook1.xls#RANGE!_ENREF_13%23RANGE!_ENREF_13) | African American | 2DM | 197 | 471 |  |  | 5974 | NPL |
| [Elbein (2009)[55]](../../../../C:%5CDocuments%20and%20Settings%5CAdministrator%5C%E6%A1%8C%E9%9D%A2%5CBook1.xls#RANGE!_ENREF_14%23RANGE!_ENREF_14) | African America | 2DM/BMI | 580 | 1293 |  |  | 5870 | NPL |
| [Unoki (2008)[56]](../../../../C:%5CDocuments%20and%20Settings%5CAdministrator%5C%E6%A1%8C%E9%9D%A2%5CBook1.xls#RANGE!_ENREF_15%23RANGE!_ENREF_15) | Japanese | 2DM |  |  | 194 | 1558 | 207,097 | *P*-value |
| [Yasuda (2008)[57]](../../../../C:%5CDocuments%20and%20Settings%5CAdministrator%5C%E6%A1%8C%E9%9D%A2%5CBook1.xls#RANGE!_ENREF_16%23RANGE!_ENREF_16) | Japanese | 2DM |  |  | 1612 | 1424 | 82,343 | *P*-value |
| [Steinthorsdottir (2007)[58]](../../../../C:%5CDocuments%20and%20Settings%5CAdministrator%5C%E6%A1%8C%E9%9D%A2%5CBook1.xls#RANGE!_ENREF_17%23RANGE!_ENREF_17) | Icelandic | 2DM |  |  | 1399 | 5275 | 313,179 | *P*-valu |
| [Wellcome Trust Case Control Consortium (2007)[2]](../../../../C:%5CDocuments%20and%20Settings%5CAdministrator%5C%E6%A1%8C%E9%9D%A2%5CBook1.xls#RANGE!_ENREF_18%23RANGE!_ENREF_18) | British | 2DM |  |  | 1924 | 2938 | 469,557 | *P*-valu |
| [Hayes (2007)[59]](../../../../C:%5CDocuments%20and%20Settings%5CAdministrator%5C%E6%A1%8C%E9%9D%A2%5CBook1.xls#RANGE!_ENREF_19%23RANGE!_ENREF_19) | Mexican American | 2DM |  |  | 281 | 280 | 88,142 | *P*-valu |
| [Scott (2007)[60]](../../../../C:%5CDocuments%20and%20Settings%5CAdministrator%5C%E6%A1%8C%E9%9D%A2%5CBook1.xls#RANGE!_ENREF_20%23RANGE!_ENREF_20) | Finnish | 2DM |  |  | 1161 | 1174 | 315,635 | *P*-valu |
| [Rampersaud (2007)[61]](../../../../C:%5CDocuments%20and%20Settings%5CAdministrator%5C%E6%A1%8C%E9%9D%A2%5CBook1.xls#RANGE!_ENREF_21%23RANGE!_ENREF_21) | mixed | 2DM |  |  | 2266 | 2412 | 82,485 | *P*-value |
| [Sladek (2007)[62]](../../../../C:%5CDocuments%20and%20Settings%5CAdministrator%5C%E6%A1%8C%E9%9D%A2%5CBook1.xls#RANGE!_ENREF_22%23RANGE!_ENREF_22) | French | 2DM |  |  | 661 | 614 | 392935 | P-value |
| [Hanson (2007)[63]](../../../../C:%5CDocuments%20and%20Settings%5CAdministrator%5C%E6%A1%8C%E9%9D%A2%5CBook1.xls#RANGE!_ENREF_23%23RANGE!_ENREF_23) | American Indians | 2DM | 140 | 121 | 300 | 334 | 80044 | *P*-value |
| [Florez (2007)[64]](../../../../C:%5CDocuments%20and%20Settings%5CAdministrator%5C%E6%A1%8C%E9%9D%A2%5CBook1.xls#RANGE!_ENREF_24%23RANGE!_ENREF_24) | Caucasian | 2DM | 307 | 91 |  |  | 66,543 | *P*-value |
| [Ng (2004)[65]](../../../../C:%5CDocuments%20and%20Settings%5CAdministrator%5C%E6%A1%8C%E9%9D%A2%5CBook1.xls#RANGE!_ENREF_25%23RANGE!_ENREF_25) | Chinese | 2DM | 64 | 126 |  |  | 355 | NPL |
| [Rotimi (2004)[66]](../../../../C:%5CDocuments%20and%20Settings%5CAdministrator%5C%E6%A1%8C%E9%9D%A2%5CBook1.xls#RANGE!_ENREF_26%23RANGE!_ENREF_26) | West Africa | 2DM | 343 | 691 |  |  | 390 | LOD |
| [Silander (2004)[67]](../../../../C:%5CDocuments%20and%20Settings%5CAdministrator%5C%E6%A1%8C%E9%9D%A2%5CBook1.xls#RANGE!_ENREF_27%23RANGE!_ENREF_27) | Finnish | 2DM | 737 | 1709 |  |  | 392 | MLS |
| [Xiang (2004)[68]](../../../../C:%5CDocuments%20and%20Settings%5CAdministrator%5C%E6%A1%8C%E9%9D%A2%5CBook1.xls#RANGE!_ENREF_28%23RANGE!_ENREF_28) | Chinese | 2DM | 257 | 385 |  |  | 388 | NPL |
| [Sale (2004)[69]](../../../../C:%5CDocuments%20and%20Settings%5CAdministrator%5C%E6%A1%8C%E9%9D%A2%5CBook1.xls#RANGE!_ENREF_29%23RANGE!_ENREF_29) | African American | 2DM | 247 | 638 |  |  | 390 | NPL |
| [Nawata (2004)[70]](../../../../C:%5CDocuments%20and%20Settings%5CAdministrator%5C%E6%A1%8C%E9%9D%A2%5CBook1.xls#RANGE!_ENREF_30%23RANGE!_ENREF_30) | Japanese | 2DM | 102 | 102 |  |  | 382 | LOD/MLS |
| [Iwasaki (2003)[71]](../../../../C:%5CDocuments%20and%20Settings%5CAdministrator%5C%E6%A1%8C%E9%9D%A2%5CBook1.xls#RANGE!_ENREF_31%23RANGE!_ENREF_31) | Japanese | 2DM/lnBMI | 164 | 256 |  |  | 414 | MLS/LOD |
| [Reynisdottir (2003) [72]](../../../../C:%5CDocuments%20and%20Settings%5CAdministrator%5C%E6%A1%8C%E9%9D%A2%5CBook1.xls#RANGE!_ENREF_32%23RANGE!_ENREF_32) | Icelandic | 2DM/BMI | 227 | 763 |  |  | 906 | LOD |
| [Busfield (2002) [73]](../../../../C:%5CDocuments%20and%20Settings%5CAdministrator%5C%E6%A1%8C%E9%9D%A2%5CBook1.xls#RANGE!_ENREF_33%23RANGE!_ENREF_33) | Australians | 2DM | 232 | 138 |  |  | 474 | LOD |
| [Wiltshire (2001)[74]](../../../../C:%5CDocuments%20and%20Settings%5CAdministrator%5C%E6%A1%8C%E9%9D%A2%5CBook1.xls#RANGE!_ENREF_34%23RANGE!_ENREF_34) | Caucasian | 2DM | 573 | 743 |  |  | 418 | NPL |
| [Ehm (2000)[75]](../../../../C:%5CDocuments%20and%20Settings%5CAdministrator%5C%E6%A1%8C%E9%9D%A2%5CBook1.xls#RANGE!_ENREF_35%23RANGE!_ENREF_35) | mixed | 2DM | 362 | 896 |  |  | 389 | NPL |
| Total |  |  | 4532 | 8423 | 42200 | 86253 |  |  |

^a^ Ped = total genotyped pedigrees; ^b^ AFF = total genotyped cases. LOD, logarithm of odds; NPL, Nonparametric multipoint linkage; MLS, maximum LOD score

Table S2. Characteristics of whole genome studies of obesity or BMI-defined obesity

| First author (year) | Population | Phenotype | Peda | Affb | Case | Control | Markers | Statistics |
| --- | --- | --- | --- | --- | --- | --- | --- | --- |
| Okada (2012)[76] | Japanese | BMI |  |  | 62,245 | | 2,178,018 | *P*-value |
| Wei (2012)[77] | European | BMI |  |  | 3,653 | | 2,474,474 | *P*-value |
| Ng (2012)[78] | African American | BMI |  |  | 1715 | | 746,626 | *P*-value |
| Malhotra (2011)[79] | Pima Indians | BMI |  |  | 1120 | | 454,194 | *P*-value |
| Jiao (2011)[80] | European | Obesity |  |  | 164 | 163 | 406,177 | *P*-value |
| Wang (2011)[81] | Caucasian | Obesity |  |  | 520 | 540 | 550,000 | *P*-value |
| Dong (2011)[82] | Caribbean Hispanic | Obesity |  |  | 1390 | | 383 | LOD |
| Croteau (2011)[83] | Filipino | Obesity |  |  | 1792 | | 2,073,674 | *P*-value |
| Speliotes (2010)[84] | European | BMI |  |  | 123,865 | | 2.8 million | *P*-value |
| Liu (2010)[85] | European | BMI |  |  | 11,53 | | 559,712 | *P*-value |
| Johansson (2010)[86] | European | BMI |  |  | 80,969 | | 305,846 | *P*-value |
| Meyre(2009)[87] | Caucasian | Obesity |  |  | 1380 | 1416 | 865,000 | *P*-value |
| Sammalisto (2009)[88] | African, European-American | BMI | 3032 | 9371 |  |  | 400 | LOD |
| Sabatti (2009) [89] | Caucasian | BMI |  |  | 4763 | | 329,091 | *P*-value |
| Liu (2008)[90] | US Caucasians | Obesity |  |  | 1000 | | 379,319 | *P*-value |
| Thorleifsson (2008)[91] | Icelanders | BMI |  |  | 25,344 | 23,190 | 305,846 | *P*-value |
| He(2008)[92] | Caucasians | BMI | 427 | 3273 |  |  | 410 | LOD |
| Ciullo (2008)[93] | Cilento | BMI/obesity |  |  | 925 | | 1122 | LOD/ZLR |
| Scuteri (2007)[94] | Caucasian | BMI |  |  | 4743 | | 362,129 | *P*-value |
| Almasy (2007)[95] | American Indian | BMI | 58 | 963 |  |  | 400 | LOD |
| Guo (2006)[96] | Caucasians | BMI | 4247 | 379 |  |  | 393 | LOS |
| Groves (2006)[97] | Caucasians | BMI | 573 | 1215 |  |  | 418 | LOD |
| Herbert (2006)[98] | Caucasians | obeisty |  |  | 694 | | 86,604 | *P*-value |
| Li (2004) [99] | Caucasians | obeisty | 260 | 1297 |  |  | 382 | NPL |
| Arya (2004)[100] | Mexican Americans | BMI | 27 | 430 |  |  | 326 | LOD |
| Heijmans (2004)[101] | Netherlands | BMI | 192 | 525 |  |  | 379 | LOD |
| Meyre (2004)[102] | Caucasians | BMI | 115 | 506 |  |  | 431 | LOD |
| Bell (2004)[103] | French Caucasians | obesity |  |  | 447 | | 400 | LOD |
| Saar (2003) [104] | Caucasians | obesity | 89 | 369 |  |  | 437 | LOD |
| Adeyemo (2003) [105] | Caucasians | BMI | 182 | 769 |  |  | 402 | LOD |
| Deng (2002)[106] | Caucasians | BMI | 53 | 630 |  |  | 380 | MLS |
| Wu (2002)[107] | mixed | BMI |  |  | 6849 | | 372 | LOD |
| Feitosa (2002)[108] | Caucasians | BMI | 718 | 4211 |  |  | 404 | LOD |
| Stone (2002)[109] | Caucasians | obesity | 64 | 1687 |  |  | 628 | NPL |
| Perola (2001)[110] | Caucasians | BMI | 247 | 614 |  |  | 350 | LOD |
| Hsueh (2001)[111] | Caucasians | BMI | 200 | 672 |  | | 357 | LOD |
| Total |  |  | 9973 | 23938 | 348,887 | |  |  |

^a^ Ped = total genotyped pedigrees; ^b^ AFF = total genotyped cases. ZLR, ikelihood ratio z-score; LOD, logarithm of odds; NPL, Nonparametric multipoint linkage; MLS, maximum LOD score

Table S3. Characteristics of whole genome studies of CAD

| First author (year) | Population | Phenotype | Peda | Affb | Case | Control | Markers | Statistics |
| --- | --- | --- | --- | --- | --- | --- | --- | --- |
| Davies (2012)[112] | European | CAD |  |  | 7123 | 6826 | 5 million | *P*-value |
| Takeuchi (2012)[113] | Japanese | CAD |  |  | 806 | 1337 | 451,382 | *P*-value |
| Barbalic (2011)[114] | African American | CAD |  |  | 362 | 2543 | - | *P*-value |
| Slavin (2011)[115] | European | CAD |  |  | 2000 | 3000 | 407,576 | *P*-value |
| Wild (2011)[116] | European | CAD |  |  | 2078 | 2953 | 608,247 | *P*-value |
| Schunkert (2011)[117] | European | CAD |  |  | 22233 | 64762 | 2.3 million | *P*-value |
| CAD Genetics Consortium (2011)[118] | European/ Asian | CAD |  |  | 15420 | 15422 | 574,919 | *P*-value |
| Wang (2011)[119] | Chinese | CAD |  |  | 230 | 230 | 440,794 | P-value |
| Reilly (2011)[120] | European | CAD/MI |  |  | 6380 | 3654 | 2.4 million | *P*-value |
| Erdmann (2010)[121] | European | CAD |  |  | 1157 | 1748 | 254,558 | *P*-value |
| Erdmann (2009)[122] | German | CAD/MI |  |  | 6990 | 8955 | 567,119 | *P*-value |
| Tregouet (2009)[123] | Caucasian | CAD |  |  | 1926 | 2938 | 500,000 | *P*-value |
| Roberts (2007)[124] | Caucasian | CAD |  |  | 322 | 312 | 72,864 | *P*-value |
| Samani (2007)[125] | Caucasian | CAD/MI |  |  | 2801 | 4582 | 377,857 | *P*-value |
| Farrall (2006)[126] | Caucasian | CAD | 2036 | 2658 |  |  | 446 | MLS |
| Samani (2005)[127] | United Kingdom | CAD | 1933 | 4175 |  |  | 416 | LOD |
| Hauser (2004)[128] | Caucasian | CAD | 438 | 493 |  |  | 395 | LOD |
| Wang (2004)[129] | Caucasian | CAD | 428 | 1613 |  |  | 408 | LOD |
| Pajukanta (2000)[130] | Caucasian | CAD | 156 | 364 |  |  | 303 | MLS |
| Francke (2001)[131] | North-Eastern | CAD | 99 | 240 |  |  | 400 | LOD |
| Broeckel (2002)[132] | Caucasian | CAD | 519 | 1406 |  |  | 394 | LOD |
| Total |  |  | 2036 | 2658 | 69828 | 119262 |  |  |

^a^ Ped = total genotyped pedigrees; ^b^ AFF = total genotyped cases. LOD, logarithm of odds; MLS, maximum LOD score
